# Rocky Mountain Spotted Fever Masquerading as Gastroenteritis: A Common but Overlooked Clinical Presentation

**DOI:** 10.7759/cureus.14438

**Published:** 2021-04-12

**Authors:** David S Braun, Ian Greenberg, Mangesh Pagadala

**Affiliations:** 1 Internal Medicine, Methodist Dallas Medical Center, Dallas, USA; 2 Hepatology, Methodist Dallas Medical Center, Dallas, USA

**Keywords:** gastroenteritis, rocky mountain spotted fever, abnormal liver function test, enteritis, fever

## Abstract

Rocky Mountain spotted fever (RMSF) is a tick-borne illness caused by *Rickettsia rickettsii*. The classic triad of fever, rash, and a recent tick bite is rarely present at diagnosis. Less known, but more common initial presentations include gastrointestinal symptoms such as anorexia, nausea, vomiting, and abdominal pain. In endemic areas, a persistent fever with gastrointestinal symptoms should prompt screening and early initiation of antibiotics to prevent the development of fulminant RMSF and its associated high mortality. This case aims to educate about the gastrointestinal and hepatic manifestations of this diagnostic enigma.

## Introduction

Rocky Mountain spotted fever (RMSF) is a potentially lethal tick-borne illness caused by *Rickettsia rickettsii*. Geographically, RMSF occurs most frequently in southeastern and midwestern states; however, cases occur throughout the contiguous United States, North America, and South America. The two principal RMSF vectors in the United States are Dermacentor andersoni (Rocky Mountain wood tick) and Dermacentor variabilis (American dog tick) [1]. RMSF multiplies within blood vessels, causing endothelial injury before spreading hematologically to affect various organs. Symptoms can include fever, rash, and tick bite; however, gastrointestinal symptoms, such as anorexia, nausea, vomiting, and abdominal pain, are more common. Death may occur in fulminant RMSF, thus prompt diagnosis and early administration of appropriate antibiotics are imperative. We present a case of RMSF at a North Texas hospital that demonstrates the diagnostic challenges associated with this illness.

## Case presentation

A 20-year-old male presented to the emergency department (ED) with three days of persistent fever, cough, and worsening sore throat. He had no prior medical conditions, no routine medications, and was taking Tylenol and Ibuprofen for symptom control. He had no recent travel history or sick contacts. He denied alcohol or illicit drug use but smoked half a pack of cigarettes daily. Vitals revealed sinus tachycardia and a fever of 39.4^o^C (103^o^F). His complete blood count and basic metabolic panel were unremarkable except for mild thrombocytopenia. Due to his symptoms, the patient was tested for COVID-19 and discharged home with instructions to practice social distancing and take Tylenol for fever. His COVID-19 test returned negative.

**Figure 1:**
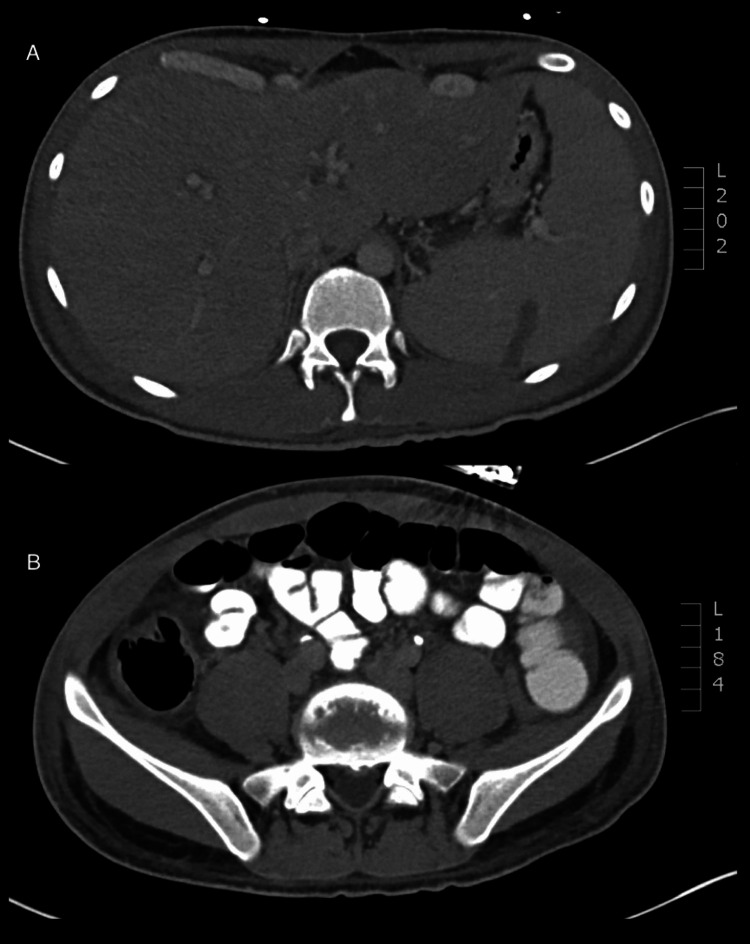
Abdominal computed tomography scan with diffuse inflammation causing the appearance of hepatomegaly and splenomegaly (A), appendicitis and colitis (B).

The patient returned to the ED five days later with persistent high-grade fever and sore throat that had progressed to odynophagia. Additionally, he had progressive right upper quadrant abdominal pain, nausea, and profuse diarrhea. An exam showed a fever of 40.1^o^C (104.2^o^F) and abdominal tenderness most severe in his right upper quadrant. Compared to five days prior, admission lab work revealed a decrease in hemoglobin (14.2 g/dL vs. 11.4 g/dL), platelet count (102 x 10^9^/L vs. 50 x 10^9^/L), and sodium (138 mEq/L vs. 129 mEq/L) with a concomitant increase in creatinine (0.7 mg/dL vs. 1.3 mg/dL). Liver function tests revealed elevated levels of aspartate aminotransferase (AST; 240 U/L), alanine aminotransferase (ALT; 247 U/L), total bilirubin (7.2 mg/dL), and alkaline phosphatase (ALP; 451 U/L). An abdominal computed tomography scan revealed appendiceal and gallbladder inflammation with surrounding edema, cecal thickening, ascites, periportal edema, and splenomegaly with infarction (Figures 1A, 1B).

**Figure 2:**
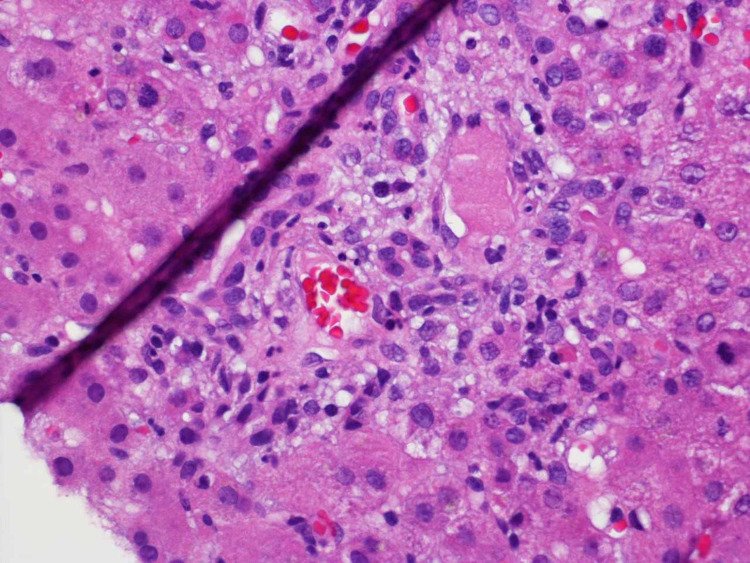
Liver biopsy in RMSF revealing a nonspecific reactive hepatitis with peri-portal inflammation and surrounding bile ductular inflammation and proliferation. RMSF - Rocky Mountain spotted fever

The patient was empirically placed on vancomycin and piperacillin-tazobactam. Hepatology and general surgery were consulted. Appendicitis was ruled out, but a hepatic sonogram showed a thickened gallbladder with surrounding edema, hepatomegaly without any focal masses, and a normal common bile duct. Magnetic resonance cholangiopancreatography revealed no biliary dilation. A liver biopsy revealed portal-based inflammation consisting predominantly of neutrophils, and mild bile ductular proliferation and inflammation (Figure 2).

Over the next three days, he had persistent abdominal pain, diarrhea, and high-grade fever. Additionally, he had worsening leukocytosis and anemia. His liver enzymes remained elevated but stable. Chronic liver disease and infectious disease work ups showed that he was negative for Epstein-Barr virus, cytomegalovirus, hepatitis A-E, HIV, Legionella, Clostridium difficile, stool ova, parasites, anti-nuclear antibodies, anti-SM antibodies, and toxicology screen.

On the fourth day, a serologic test for RMSF IgM antibodies was positive (titer = 1:1024; <1:64 = negative). Upon further questioning, no history of a tick bite or any epidemiological risk factors were elicited. The patient’s antibiotics were immediately changed to doxycycline. Twenty-four hours after the antibiotic change his fever resolved and gastrointestinal symptoms improved. The patient was discharged home on a course of doxycycline. At a two-week follow-up visit, his symptoms had resolved and his liver enzymes, blood counts, and electrolytes had returned to normal.

## Discussion

RMSF is an uncommon disease that remains a diagnostic challenge for physicians. The name itself is a misnomer, as RMSF has been reported throughout the United States, Canada, and Mexico. Fever, rash, and tick bite are noted in approximately 60% of RMSF patients; however, less than 3% have these symptoms in the first 72 hours, up to 11% do not develop a rash [1-6], and approximately 40% do not report a history of tick bite [1,7]. Gastrointestinal symptoms, such as anorexia, nausea, vomiting, and abdominal pain, are the more prominent features in up to 80% of RMSF patients; up to 45% of patients report diarrhea [8].^ ^The gastrointestinal symptoms often precede the appearance of a rash by more than 72 hours and often mimic other pathologies, leading to diagnostic delay [4,6]. Consideration of erroneous diagnoses such as acute abdomen secondary to cholangitis or appendicitis, can lead to unnecessary workups that contribute to unnecessary risk and costs [2,5,8,9]. Liver involvement in RMSF is also common, most frequently manifesting as AST and ALT elevation [3,10]. Jaundice is seen less frequently but may portend a poorer prognosis [8].

The pathogenesis of gastrointestinal and hepatic involvement is not fully understood. RMSF putatively causes inflammation, edema, and ischemia in the gastrointestinal tract nerves [2]. Pathological specimens demonstrated Rickettsial vascular invasion of the resected visceral organs, suggesting a vasculitic process [2,8,9]. Similarly, liver biopsies revealed infection of the endothelial lining and liver sinusoids as well as periportal inflammation [11].

RMSF diagnosis is often based on epidemiological factors and symptoms because serologic tests are usually not available in the acute setting [12]. Also, IgM and IgG antibodies may be falsely negative within the first week of illness [13]. Therefore, antibiotics are generally initiated empirically and serology is used as a confirmatory test. The treatment of choice for RMSF is doxycycline for at least seven days [14,15].^ ^The majority of patients experience improvement in clinical signs and symptoms within 72 hours. Delayed RMSF treatment can lead to an increased mortality rate of up to 25% [13,14].

## Conclusions

In summary, numerous factors may hinder RMSF diagnosis, including geographical misconceptions based on the name, non-specific presentation, under-recognition of gastrointestinal symptoms, and unavailable serologic tests. As a result, expensive workups and invasive procedures may be pursued and appropriate treatment delayed. As seen in our case study, the current prevalence of COVID-19 adds yet another misleading variable. It is imperative that clinicians be educated about the early manifestation of RMSF and consider it among the differential diagnoses in a patient with fever, gastrointestinal symptoms, and hepatic involvement regardless of geographical location. If there is clinical suspicion for RMSF, doxycycline must be started immediately to significantly reduce mortality.
